# “Mushrooms (and a cow) are A Means of Survival for Us”: Dissimilar Ethnomycological Perspectives among Hutsuls and Romanians Living Across The Ukrainian-Romanian Border

**DOI:** 10.1007/s00267-022-01619-6

**Published:** 2022-03-30

**Authors:** N. Stryamets, G. Mattalia, A. Pieroni, R. Sõukand

**Affiliations:** 1 Ca’ Foscari University of Venice, Via Torino 155, Venice, Italy; 2 Nature reserve “Roztochya”, Sitchovuh Strilciv 7, 81070 Ivano-Frankove, Ukraine; 3 University of Gastronomic Sciences, Piazza Vittorio Emanuele 9, 12042 Pollenzo, Bra (Cn) Italy; 4 Medical Analysis Department, Tishk International University, 44001 Erbil, Iraq

**Keywords:** Bukovina, Carpathian Mountains, fungi, ethnomycology, Hutsuls, rural areas

## Abstract

Sustainable forest management highlights the multipurpose use of all forest resources, including the use of wild mushrooms, by a variety of forest users and especially for rural livelihoods. To achieve sustainable forest management, among others, decision-makers and forest managers need to identify the important elements for the livelihoods of local communities dependent on forests. Therefore, our aim is to analyse the importance of contemporary use of wild mushrooms for daily livelihoods in rural areas of the Carpathian Mountains by comparing two ethnic groups, Hutsuls and Romanians, living in a similar ecological environment and formerly belonging to the historical region of Bukovina, but currently split by the border between Ukraine and Romania which have different governments and economic situations. One hundred and twenty-one face-to-face semi-structured interviews were conducted in the summers of 2018 and 2019. We compared the Traditional Ecological Knowledge (TEK) of Romanians and Hutsuls living, respectively, in lowlands and mountain areas on both sides of the border. Our results demonstrated the homogenous use of mushroom species for cultural purposes (e.g. ritual foods). Yet, we detected a remarkable difference in the role mushrooms play in providing income: Hutsuls in Ukraine use forest products as a main (rarely additional) source of income, while Romanian Hutsuls use them solely as additional income. Romanians on both sides considered mushrooms mainly as food and did not sell them (probably due in part to less abundance in the area). We also documented the fear of local residents that forest management and protected areas could suppress the right to collect wild mushrooms. The use of mushrooms is an important aspect of local TEK and needs to be considered as a part of sustainable forest management and as a means of poverty reduction in the region.

## Introduction

“To all people of Ukraine who fight so bravely for the freedom of all of us”.

In the context of sustainable development, there is a need to reduce the impact of food with a high ecological footprint (Hickey et al. 2016) and to diversify the food basket. One possible solution, for those communities living in the vicinity of woodlands, is the use of forest foods generally included among non-wood forest products (NWFPs) (FAO, 2015). Indeed, the FAO and IPBES highlight that the use of forest products, such as mushrooms, could potentially contribute to food security and income–generation, especially in rural, marginal remote areas (FAO 1999; Karki et al. 2017). Moreover, Garibay-Orijel et al. (2009) claimed that the use of wild mushrooms could be integrated into sustainable forest management models for indigenous and local communities. Actually, rural communities often do consider wild mushrooms not only as a source of nutrition, but also as crucial ingredients of local diets for their aroma and taste (Rotola-Pukkila et al. 2019), in addition to being a source of income (Hickey et al. 2016, Tibuhwa 2013, Voces et al. 2012) especially among the poorest sections of society (Shackleton and Gumbo 2010, Stryamets et al. 2015). A number of studies have demonstrated that wild mushrooms are one of the most important wild forest products used in different EU countries (Boa 2004, Prokofieva et al. 2017, Schulp et al. 2014, Stryamets 2016). However, scholars have found that cultural as well as ethnic belonging highly influence the use of wild mushrooms (Boa 2004, Prokofieva et al. 2017). For example, only 3% of Danish people pick mushrooms compared to 40% for the Finnish (Prokofieva et al. 2017, p. 147). Indeed, the rate of mushroom use results from different attitudes towards mushroom picking, for instance, being either mycophilic or mycophobic (Peintner et al. 2013). Furthermore, Colfer (2012) explained that reasons for using mushrooms differ in Europe because of the divergent roles they play. While in developed European countries they have become fashionable cuisine following the growing demand for (mountain) forest products (Elbakidze and Angelstam 2007), in developing countries they still play an important role in everyday cooking and, as with other forest products, promoting diet diversification and economic insurance (Nerfa et al. 2020). In regard to ethnomycological knowledge transmission, Garibay-Orijel et al. (2012) studied gender as one of the key variables to the transmission of local mycological knowledge, with women as the main keepers of this knowledge. Another study carried out in Poland indicated that it is an important family activity and that schools have less influence on local ethnomycological knowledge than books (Łuczaj and Nieroda 2011). Nevertheless, no studies have addressed the impact of different policies and governance systems on the use of wild mushrooms among culturally homogenous communities. Indeed, cross-border studies could greatly improve our understanding of how different “national/state” governances or policies affect the use of NWFPs. In addition, they can provide important insight into how the use of wild products varies according to cultural preferences (Pacheco-Cobos et al. 2015) in similar ecological contexts.

Recently, Bukovina, a historical region of Eastern Europe, has served as a “study lab” for a number of cross-border and cross-cultural ethnobotanical studies on wild foods and medicinal plants, including NWFPs (Mattalia et al. 2020, 2021, Pieroni and Sõukand 2017, Sõukand and Pieroni 2016, Stryamets et al. 2021, Stryamets et al. 2021). Bukovina is indeed an ideal case study for cross-border and cross-cultural ethnobiological investigations because it is a multicultural and multi-ethnic region which was united until the 1940s, at which time it was divided between present-day Ukraine and Romania. The use of wild mushrooms for food has been documented in Eastern Europe (Keča et al. 2013, Łuczaj et al. 2015, 2013, 2012, Pieroni and Sõukand 2017, 2018 Schulp et al. 2014, Sõukand and Pieroni 2016), but only a few scientific and focused studies have been conducted in Romania and Ukraine. There have been some descriptive studies, mostly in local languages, on wild edible mushrooms in Ukraine (Dudka 2003), but some of these were conducted nearly a century ago (Pilát 1926). During Soviet times, a number of studies were conducted after 1970 which together described more than 200 species of mushroom and 67 recipes on how to cook them (Dudka and Wasser 1987, Zerova and Wasser 1972). More recently, an ethnolinguistic and ethnomedicinal study of the mushrooms used in Northern Ukraine was carried out by Prus (2010), which included very detailed descriptions of the use of *Boletus edulis* and *Amanita muscaria*.

As in Ukraine, in Romania there was a wealth of popular literature on mushroom picking in the 1970s and the 1980s (Eliade and Toma 1977, Mateescu 1983, Rimbu 1982), including a guide book on how to cook wild and cultivated mushrooms (Poleac 1981); however, a very limited number of recent studies have been conducted to document and discuss this phenomenon. Vasile et al. (2017) analysed the potential and limits of the current Romanian wild mushroom sector and indicated Suceava County (corresponding to former South Bukovina) as the most important area of the country for the mushroom market. Other recent studies have documented mushroom use in specific areas of Romania such as Maramureş (Łuczaj et al. 2015), Dâmbovița (Georgescu et al. 2017), and Suceava (Tanase and Grudnicki 2003), and Cîrnu and Nichiforel (2014) included mushroom picking as an important tourist resource for the Bukovina region. Bukovina is also ideal for studying ethnomycology because both Romania and Ukraine are considered mycophilic countries (Peintner et al. 2013, Wasson and Wasson 1957), as they widely use wild mushrooms in cultural, social and culinary practices (Karki et al. 2017).

While several researchers (e.g. Wiersum 2017, Newton et al. 2016) have advocated for a better understanding of the role wild mushrooms play in forest-dependent communities, this issue remains largely underexplored. Therefore, the aim of this study is to analyse the contemporary use of wild mushrooms for daily livelihoods in rural areas of the Carpathian Mountains by comparing two ethnic groups, Hutsuls and Romanians, living in a similar ecological environment and formerly belonging to the same historical region of Bukovina, but currently split into Ukraine and Romania which have different governments and economic situations. To this end we:

- recorded traditional ecological knowledge (TEK) related to the food uses of mushroom taxa among four communities in Bukovina (specifically Hutsuls and Romanians living in Romanian Bukovina, and Hutsuls and Romanians living Ukrainian Bukovina), and
- compared the uses and perceptions of edible mushrooms and the transmission of ethnomycological knowledge in a cross-cultural (Hutsuls vs Romanians) and cross-border (Ukraine vs Romania) analysis.

The study design of contrasting governance and economic systems on the one hand, and ethnicities on the other (and the interaction of the two as a third dimension), may allow us to contribute to significant debates that advance ethnobotanical theory. Our first hypothesis is that the appurtenances of an ethnic group define the use of wild mushrooms. Our second hypothesis is that the economic situation in a region influences the use of wild mushrooms.

## Methods

### Study Areas

#### Bukovina region

**Fig. 1:**
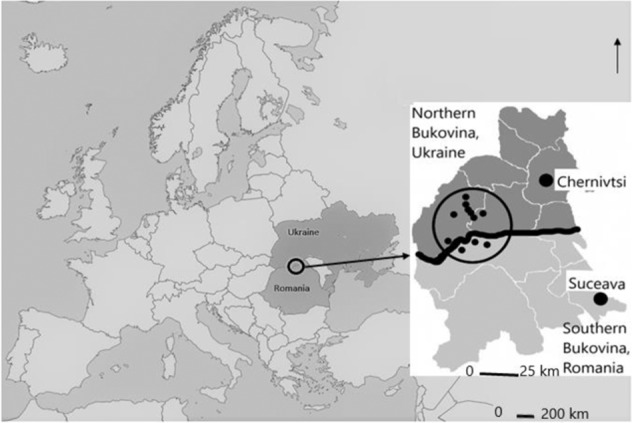
Map of the region

Bukovina is a historical region divided between Ukraine (corresponding to Chernivci Region) and Romania (approximately corresponding to present-day Suceava County) partially located in the Carpathian Mountain region, on the banks of the Prut and Siret rivers (Fig. 1). The region’s complex history, such as belonging to various governance systems (Turkish Empire, Austro-Hungarian Empire, Kingdom of Moldova, Kingdom of Romania, and divided between Ukrainian SSR and the Republic of Romania), has led to a unique population structure and natural resource use (Leahu et al. 2019, Musiienko 2007, Vasylova 2008).

**Fig. 2:**
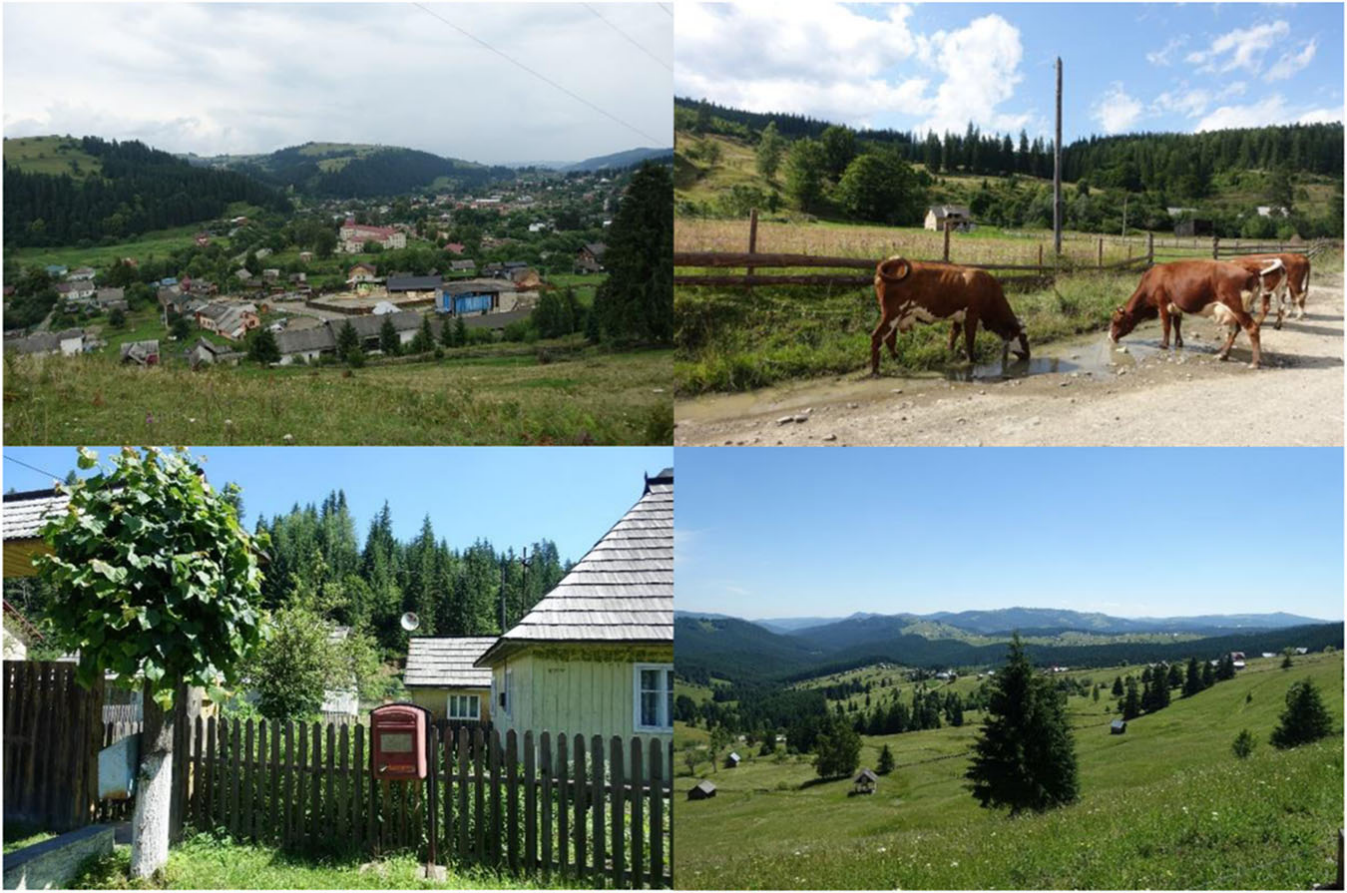
Hutsul landscapes. Top row: Hutsul settlements in Ukrainian Bukovina; bottom row: Hutsul settlements in Romanian Bukovina. Photos by N. Stryamets, summers 2018–2019

**Fig. 3:**
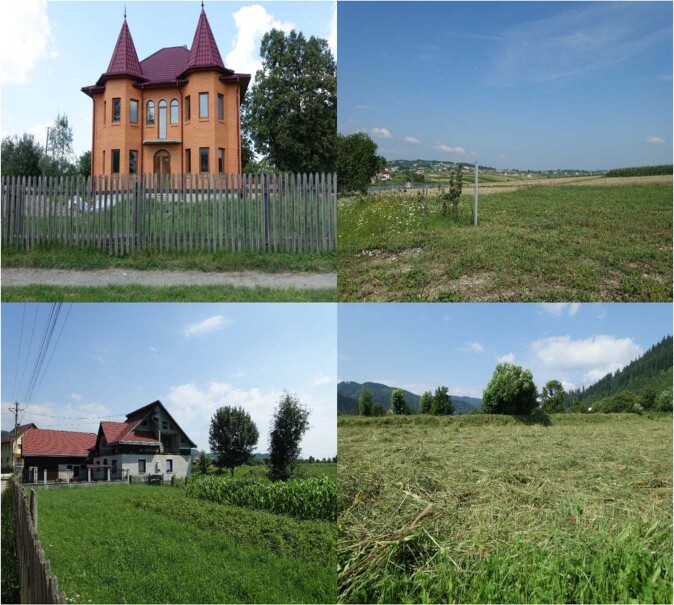
Romanian landscapes. Top row: Romanian settlements in Ukrainian Bukovina; bottom row: Romanian settlements in Romanian Bukovina. Photos by N. Stryamets, summers 2018-2019

One Bukovina – two ethnos in one territory divided by a border between two countries. On both sides of the border, Hutsuls live in the mountain regions (500–1400 m a.s.l.), while Romanians inhabit lower altitudes characterized by hilly landscapes (Figs. 2, 3). The two groups are characterised by different social conditions (Hutsuls are more isolated, with little arable land and a wide availability of forest products), ecological conditions (Romanians living in lowlands have warmer climatic conditions and a wider availability of arable land) and economic conditions (since Romania joined the EU the level of salaries and pensions has grown, and for Ukrainian Romanians there is the possibility of obtaining Romanian passports and then freely moving to other EU countries for seasonal or permanent employment) (Table 1). For all groups, houses are often accompanied by small-scale gardens in which fruit trees (apples, cherries, plums, etc.) and vegetables (potatoes, cabbage, carrots, onions, garlic, etc.) are grown.

**Table 1:** Government system indices for the two countries

Indices	Northern Bukovina, Ukraine	Southern Bukovina, Romania
Access to the resource	Free for personal consumption Permit for commercial purposes	Free for personal consumption Permit for commercial purposes
Forest ownership	100% state-owned	Private and state-owned
Unemployment		5%
	8%*,	
	Official employment rate: 58%	
Average salary (as of 2018)	6100 UAH (~200 EUR/per month)	2137 RON (~442 EUR/per month)
Average pension (as of 2018)	1700 UAH (~59 EUR/per month)	500 RON (~103 EUR/per month)

^*^Due to a complex system of bureaucracy, it is time consuming to become “officially unemployed” with social help from the state. Therefore, unemployment is 8% but the official employment rate among people 14–70 years old is 58%

##### Northern Bukovina (Ukraine)

Chernivci Region, corresponding to Northern Bukovina, was created in 1940 and is geographically formed by the mountain region and lowlands. The mountain relief is formed by the Pokutsko-Bukovinskyh Carpathians, together with the ridge of the Yalovichorskih Mountains, with the highest peak at 1574 m a.s.l. The rest of the territory is formed by the Khotynska upland. The mountain region is mainly covered by forests (63%) of Norway spruce (*Picea abies* (L.) H.Karst.), beech (*Fagus sylvatica* L.) and silver fir (*Abies alba* Mill.). The flora consists of more than 2,000 species (Anon, 2018). The region is inhabited by 906,000 people and population density is 111 people per sq. km (Anon, 2018).

Putyla District is located in the SW area of the Chernivci region, where most Hutsuls live (Table 2). It is a rural region consisting of the small central town of Putyla (3,400 inhabitants) and 32 villages. The administrative centre of the Chernivci region is only 120 km away, but due to the poor state of the concrete road the journey is time consuming. Romanians live in the region close to the Romanian border, formed by the town of Storozenets, the small town of Krasnoijlsk and 37 villages.

**Table 2:** Description of the four study regions

Hutsuls living in the mountain areas of Ukraine (U-HU)	Romanians living in the lowlands of Ukraine (U-RO)
• Negative population grow rate	• 100,000 inhabitants
• 26,000 people, rural population	• Population density of 84.3 people per sq. km.
• Population density of 29 people per sq. km.	• Nearly 37% of the population are Romanians (based on the National Census; Anon, 2018), who live in villages close to the Romanian border, including Krasnoijilsk, Chudey, Izhivci, Stara Krasnoshora, Nova Krasnoshora and Kypka (Fig. 3).
• In the district, there are 54 large farms and more than 6000 small-scale farms, a hospital and 4 clinics.	• In the region, there are 51 schools, 47 libraries and 9 hospitals, as well as 15 industrial enterprises.
• Hutsuls practice natural animal husbandry with sheep, cows, goats and horses.	• Working migration to the European Union for 3–6 months per year was mentioned by interviewees as an important source of income.
• Emigration to richer regions of Ukraine, as well as to Poland, Italy and the Czech Republic.	• Supplementary small-scale farming was also present in the region, providing vegetables and fruits.

Hutsuls living in the mountain areas of Romania (R-HU)	Romanians living in the lowlands of Romania (R-RO)
• Our research was carried out in three villages: Brodina, Ulma and Izvoarele Sucevei.	• The interviews among Romanians in Romania were conducted in the municipality of Straja, which is located next to Brodina (Fig. 3).
• Population density is low at 15–20 inhabitants per sq. km.	• It has 5000 inhabitants and lies at an altitude of 500 m a.s.l.
• Average altitude is around 600 m a.s.l. but can reach as high as 1000 m a.s.l.	• Population density is 113 inhabitants per sq. km.
• The main activities of this area are forest exploitation, producing an important share of Romanian lumber, although fewer and fewer people are now being employed in this sector, and small-scale farming.	• Many interviewees reported a consistent migration of local inhabitants to other European countries, especially after 2007 when Romanians obtained the right to freely move within the Schengen Area.
	• The main activities of this area are small-scale farming.

##### Southern Bukovina (Romania)

In Suceava County, more than 300,000 people live in towns, while the remaining 404,000 live in rural settlements. Population density is comparable with the Ukrainian data, especially that of Storozenetskyi District, at 82.6 people per sq. km. Half of the county’s land surface is covered by forests mainly of *Fagus sylvatica* and *Abies alba* but also *Carpinus betulus* and *Acer platanoides*. The region consists of some plain areas, as well as some mountain areas, including Pietrosu Peak which reaches 2100 m a.s.l. Hutsuls of Romanian Bukovina (Table 2) mainly live in eight mountain villages close to the Ukrainian border (Fig. 2). Brodina is the main village, with a population of approximate 3000 inhabitants dispersed across ten parishes. The closest administrative centre is Rădăuţi, which can be reached in 60–90 min depending on the village. We interviewed Romanians living in the municipality of Straja, which borders Hutsul municipalities. Connection routes and a less scattered system of dwellings allow the inhabitants of Straja to be relatively well connected to the administrative centre of Rădăuţi.

### Data collection and analysis

#### Data collection

A total of 121 face-to-face semi-structured interviews with local inhabitants were conducted in the summers of 2018 and 2019. In addition, 7 interviews were conducted in Kryvorivnya, the capital of Hutsul ethnic lands, to obtain information regarding the use of mushrooms away from the border. We pseudorandomly selected Romanian and Hutsul interviewees. The interviewees were approached in the street, parks and public spaces, near houses and gardens, in libraries and shops, and at bus stops. We also interviewed farmers living outside the villages. In total, we interviewed 31 Hutsuls and 30 Romanians in Ukraine, and 30 Hutsuls and 30 Romanians in Romania. The interviews were conducted in Ukrainian, Russian and Romanian depending on the preference of the respondent (Romanians were questioned with the help of a field assistant, who was a native speaker and knew the local dialect); although sometimes Hutsuls answered in their local language. The samples were conducted by first and second author with the same interview structure. The open questions on the use of wild mushrooms for food (and also medicine, rituals and cultural purposes − data which are elaborated in other publications), as well as past uses and knowledge transmission patterns were part of a larger study. The DiGe research project explores the use of wild products for a variety of purposes (food, medicine, cultural uses, ethnoveterinary medicine and others) and compares those uses cross-culturally and cross-ethnically in historical areas of Eastern Europe that have been divided by borders during the last century. Interviews lasted from 20 minutes to 2 h. The interviewees were given complete freedom to talk about the subject. However, questions related to money issues were proposed to the interviewee and only upon their approval were the questions asked (as is common in remote areas these issues are sensitive and our connection to the tax authorities and police was discussed). We strictly followed the ethical guidelines prescribed by the International Society of Ethnobiology (International Society of Ethnobiology 2008), and the study protocol was approved by the Ethical Committee of Ca’ Foscari University of Venice.

Participatory observation was performed at markets and at mushroom collecting points in both study areas. Markets were visited on specific “market days” (often once per week, changing day in each village). The notes on the varieties of sold mushrooms, types of preparation and prices were documented. In each location one collection point was visited.

The mushroom specimens were collected in 2019, but, unfortunately, we could not collect all the taxa mentioned as voucher specimens, as 2018 and 2019 were extremely unproductive in terms of mushroom yield, and 2020 fieldwork was limited due to the COVID-19 crisis. The Ukrainian specimens are stored at the “Roztochya” Nature Reserve, while the Romanian specimens are stored at Ca’ Foscari University of Venice. In this study, we focused on the higher mushrooms (Basidiomycota), using the term “mushroom” to refer to the fruiting body called sporocarps, common to many species of fungi, which are used to store and release spores into the environment, typically in the form of a rounded cap on a stalk. We focused on saprobic and symbiotic mushrooms as well as parasitic ones (e.g., *Armillaria mellea*). In both study areas, the term ‘mushroom’ (Ukrainian: ‘Гриби’ (gryby), and Romanian: ‘ciuperci’) was used for both edible and poisons species. Fungi nomenclature followed the Index Fungorum (http://www.indexfungorum.org/).

A general literature search was carried out employing the terms “use of mushrooms in Ukraine”, “use of mushrooms Eastern Europe”, “use of mushrooms in Romania”, “wild mushrooms Europe”, “sustainable forest management and mushrooms”, and “edible fungi Eastern Europe”, as well as these same terms in Ukrainian, Russian and Romanian languages. The retrieved references were then evaluated via their abstracts, and if considered relevant the whole document was read and analysed. The field notes, as well as collected relevant documents, including reports, newspapers and web-sources, were analysed to complete the data and enable data triangulation.

#### Data analysis

The information gathered from the interviewees was entered into an Excel spreadsheet in the form of detailed use reports (DUR), where each interviewee mentioned the use of mushrooms and their preparation (e.g., fresh mushrooms used for stew with sour cream).

On the basis of this database, we calculated the cultural significance index (CSI). The methodology developed by Pieroni (2001) for food uses of wild plants was adapted to calculate the Cultural Significance Index for food uses of mushrooms:

$$CSI_m = QI\,x\,AI\,x\,MFFI\,x\,TSAI$$

where QI is the frequency of quotation, AI is the availability index, MFFI is the multifunctional food use index, and TSAI is the taste score appreciation index (Table 3).

**Table 3:** Indexes and values following Pieroni (2001)

Availability Index (AI) Categories	Multi-Functional Food Use Index (MFFI) Categories		Taste Score Appreciation Index (TSAI) Categories	
Availability/Index value	Usage/	Index value	Taste/Index value	
Very common 4.0	Raw, as snack	0.5	Very poor	1
Common 3.0	Raw, in salads	1.5	Poor	2
Middle 2.0	Fried with sour cream	1	Fair	3
Rare 1.0	Boiled	1	Tasty	4
	Boiled, then stewed or fried	1.5	Very good, very tasty	5
	Soups (mixtures)	0.75		
	Stewed	1		
	Fermented, salted	1		
	Marinated	1		
	Frozen	0.75		
	Pickled	1		
	Various foods	0.5		
	Dried	1		
	Salad	1		
	Fried	1		

We did not include the parts used index (PUI), as the fruiting body of mushrooms was nearly always used, (see also Alonso-Aguilar et al. 2014). The food-medicinal role index (FMRI) was also not included in the equation as mushrooms were not considered medicinal.

Ukrainian and Hutsul names were transliterated using the system adopted by the Cabinet of Ministers of Ukraine 2010 (https://slovnyk.ua/translit.php). RAWGraphs 2.0, an open-source tool to visualize data, was used to create graphs of the types of mushroom preparations used by interviewees in both areas. We used alluvial diagrams for representing the data.

#### Sample description

**Fig. 4:**
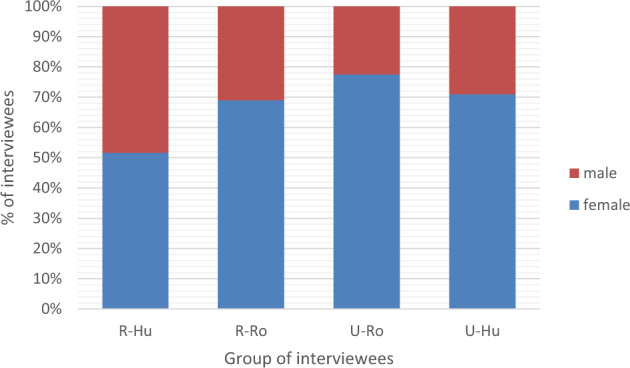
Gender distribution per group of the interviewees

**Fig. 5:**
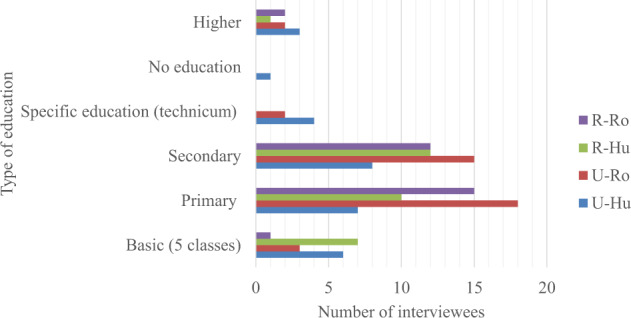
Education level per group of interviewees

The average age of the interviewees was 61 years old, and the gender ratio was 25% men and 75% women in Ukrainian Bukovina and 37% men and 63% women in Romanian Bukovina (Fig. 4). In both areas women were more available to speak with us. In all four groups, the majority of interviewees were retired, including 45% of Hutsuls and 60% of Romanians in Ukraine, as one of the requirements of the DiGe project is to interview people who have permanently lived in the area for over 40 years. Small-scale farming was mentioned as a major occupation, and some of the interviewees worked in local schools, shops and libraries. The majority of interviewees in both parts of Bukovina had primary and secondary education (Fig. 5), and only up to 10% had received a higher education.

## Results

Overall, all but two interviewees used wild mushrooms. The most used taxa, named by the majority of respondents in all four groups, were *Boletus edulis, Russula* spp., *Armillaria mellea, Cantharellus cibarius* and *Lactarius deliciosus*. The most popular taxon (see Table 4) was *Boletus edulis*, considered by all four groups of interviewees as the tastiest mushroom. In total, 19 taxa belonging to 10 families were recorded, along with three folk taxa which were not identified, as specimens could not be collected as there was no yield that year (2019).

**Table 4:** List of mushroom species used by the four groups of interviewees in Bukovina

Taxa	Local name	Local name (transliteration)	Frequency of use				Food use
			Ukraine		Romania		
			Romanians	Hutsuls	Hutsuls	Romanians	
*Agaricus* spp. Agaricaceae	Șampinioni, печериці	pecherytsi	3	1	0	0	Stew
*Armillaria mellea* (Vahl) P. Kumm., 1871 Physalacriaceae	Опеньки, підпеньки, піпінчі, Pipinci, Popinci, Ghebe, popenci	Openky, pidpenky, pipinchi	22	18	14	22	Marinated, boiled, fermented, salad
*Boletus edulis* Bull., 1782 Boletaceae	Білий, боровик, гриб, хриб, білий гриб, королівський, Hribi	Bilyi, borovyk, hryb, khryb, bilyi hryb, korolivskyi	33	31	24	22	Marinated, dried, frozen, fried, salted
*Boletus pinophilus* Pilát & Dermek, 1973 Boletaceae	Білі боровики	bili borovyky	0	1	0	0	Soup
*Cantharellus cibarius* Fr., 1821, Cantharellaceae	Лисички, лісічки, свічки, Galbiori, Gălbiori	Lysychky, lisichky	12	18	18	11	Fried, boiled, dried, frozen
*Catathelasma imperiale* (P. Karst.) Singer, 1940 Tricholomataceae	Гордубан	Horduban	0	1	0	0	Various foods
*Cortinarius caperatus* (Pers.) Fr., 1838 Cortinariaceae	Țigani, черный цыган	chernyi tsyhan	7	0	0	0	Dried, various foods
*Lactarius deliciosus* (L.) Gray, 1821 Russulaceae	Рижики, Рижі, Râșcov, Opintici	Ryzhyky, Ryzhi	20	13	11	27	Salted, fermented, marinated
*Lactarius resimus* (Fr.) Fr., 1838, Russulaceae	Грузді	Gruzdi	0	2	0	0	Fermented, salted
*Leccinum aurantiacum* (Bull.) Gray, 1821 Boletaceae	Підосичник	pidosychnyk	3	4	0	0	Dried, various foods, frozen, soup, stew
*Leccinum scabrum* (Bull.) Gray, 1821 Boletaceae	Козарі, казарики, підберезники	Kozari, kazaryky, pidbereznyky	10	7	1	0	Dried, marinated
*Morchella* spp. Morchellaceae	Zbârciog		0	0	3	0	Various foods
*Morchella esculenta* (L.) Pers., 1801 Morchellaceae	Шушерепки	Shusherepky	0	1	0	0	Various foods
*Pleurotus ostreatus* (Jacq.) P. Kumm., 1871 (Pleurotaceae)	Păstrăv fagului, Pastru		0	0	2	1	Boiled and seasoned
*Ramaria botrytis* (Pers.) Bourdot 1894 Gomphaceae	Creasta cocoșului		0	0	2	5	Various foods
*Ramaria formosa* (Pers.) Quél., 1888 Gomphaceae	Щітки	shchitky	0	3	0	0	Stew
*Russula azurea* Bres., 1882 Russulaceae	Голубінки сині	holubinky syni	1	0	0	0	Fried, fresh
*Russula* spp., Russulaceae	Сироїжки, голубінки, голубелі, Hulubți, Golubițe, Hulubițe	Syroizhky, holubinky, holubeli	21	22	16	7	Fresh, fried, boiled, pickled
*Russula virescens* (Schaeff.) Fr., 1836 Russulaceae	Голубінки зелені	holubinky zeleni	2	0	0	0	Fresh, salted, fried
*Suillus luteus* (L.) Roussel, 1796 Suillaceae	Маслюки	masliuky	0	1	0	0	Soup
*Folk taxa*	Свинське вухо	Svynske vyho	0	2	0	0	Fermented, salted
*Folk taxa*	Сірники	Sirnyky	0	2	0	0	Marinated, dried
*Folk taxa*	Tsarskiy		1	0	0	0	Various foods
*Folk taxa*	Cararuși					3	Various food

**Fig. 6:**
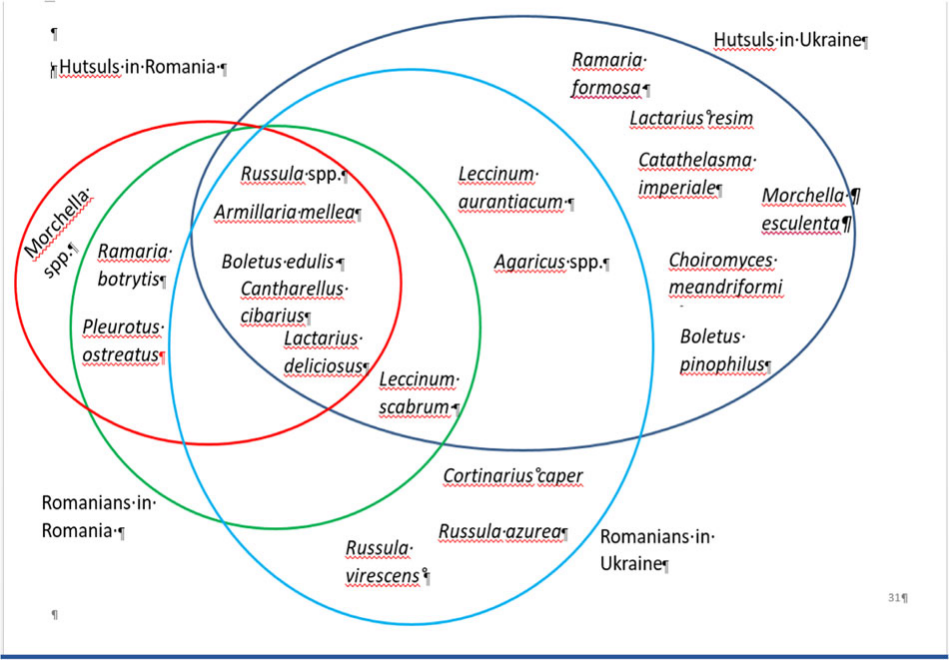
Wild mushroom taxa used by the four groups in Bukovina

Hutsuls in Ukrainian Bukovina used 16 taxa belonging to 10 families (including a variety of *Russula* species and two folk taxa), while Hutsuls in Romanian Bukovina named 9 taxa. Romanians living in Ukraine and Romania named 13 and 7 (plus one folk taxa) taxa, respectively (Fig. 6).

**Fig. 7:**
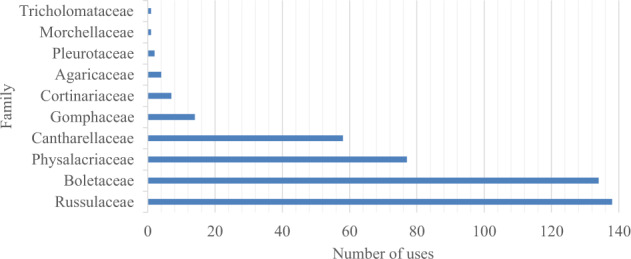
Number of uses according to taxonomic family

Russulaceae was the most named family with 138 DUR and five used taxa, including the “голубінки” - “golubinky”, or *Russula* spp., group which has 16 species in the region. The second most used family was Boletaceae with 134 DUR (Fig. 7).

The mushroom picker profiles in our case studies included both genders and all age groups. Men and women in all 4 groups collected wild mushrooms similarly. “*My husband is a real “Grybnyk” [a “mushroomer”, a person who loves to collect mushrooms, knows the habitats and species], he loves it, yesterday he collected a lot of Boletus and he pickled them*”, explained a Hutsul woman living in Ukraine. The age of the interviewee also had no effect on the use of mushrooms. Among the interviewees, children as well as elderly individuals (who can still walk in the forest) were also named as collectors. During one interview, an elderly Hutsul couple in Ukrainian Bukovina explained that their basket of mushrooms (approximately 10 kg) was collected by their grandchildren, 10 and 12 years old: “*those white mushrooms [Boletus] were collected today by our grandkids for sale*”. Only two elderly women mentioned that nowadays for them it was too difficult physically to collect mushrooms; however, in one case her son was doing it for her, while the other woman explained that she now buys mushrooms instead of collecting them herself. In villages, where locals always have other, and often hard, work to do, mushroom harvesting was considered a leisure activity and recreational time. Indeed, the interviewees expressed a positive attitude toward mushrooms and mushroom picking activities. The amount of time spent picking mushrooms depends on the yield but is often more than 3 or 4 h. The early morning is considered the proper time for picking mushrooms. Interviewees explained that when the mushroom season “starts” [when they hear that mushrooms have been found in the forest by a neighbour, which means that mushrooms have appeared in the woodlands], all other activities are postponed, and family members go to forest to collect forest goods.

### Wild Mushrooms as Food Among Hutsuls and Romanians Living in Ukrainian Bukovina

Hutsuls named only *Boletus edulis* as a proper mushroom for use, referring to them as “The Mushroom” or “Real Mushroom”, despite the richness of edible mushrooms in the Carpathian Mountains. Indeed, *Boletus* was claimed by interviewees to be the tastiest mushroom, and all other mushrooms were collected only if there was no yield of *Boletus edulis*. *Russula* species were also valued as they taste like meat and are a supplement to the local cuisine when meat is not available. The interviewees explained that forest products make a great addition to the local diet, especially in times of food scarcity. Historically, the scarcity of food (there are not enough arable lands in the mountain regions) has made locals dependent on forest products, including wild mushrooms. The interviewees explained that there are vast quantities of mushrooms in the forest, and one can easily collect 10–15 kg of mushrooms per day in a good season. That is why they were widely used as a supplementary food.

Preparation techniques included the freezing of fresh or boiled mushrooms and drying in a shaded place or in an oven or stove. Almost all mushrooms should be pre-cooked (boiled), rinsed and then cooked. There is a strong belief that boiling removes the poison from mushrooms. Only the mushrooms called ‘*сироїжки*’ – ‘fresh eating’ mushrooms (*Russula* spp.) – were reported to be sometimes eaten without pre-cooking. In most cases it is women’s work to cook mushrooms. Four Hutsul women were cleaning mushrooms while being interviewed, and they expressed the opinion that it is much harder to clean them than to pick them in the forest.

*Boletus* mushrooms were “*cooked in a pan with some garlic, onion and oil; that is it – very tasty. This recipe is good for the fasting period. At other times sour cream is also added*,” explained a middle-aged Romanian man (during the fasting period dairy products are also forbidden). Soups are generally popular in the cuisine of Hutsuls, and so soups with the addition of dried wild mushrooms were named. Stews with fresh or dried mushrooms, garlic, onions, sour cream and a variety of side dishes like pasta, potatoes and meat were also popular. The cooking recipes were transmitted from grandparents and parents to younger generations.

Hutsuls explained that the different types of mushroom preserves, such as marinated, salted and dried, have different uses. The most widely used mushroom taxa for winter preserves were *Boletus edulis* and *Armillaria mellea*. Fermented cabbage and boiled *Armillaria mellea* were used in recipes for salting*. Lactarius deliciosus* and sometimes *Russula* spp. were also salted and fermented. Since the availability of freezers after the year 2000, the fastest and easiest way to preserve mushrooms has been freezing. The drying of mushrooms was named by interviewees as a “traditional” use, as their grandparents used to do it. “*My grandfather kept dried mushrooms in cotton bags in the gorushe (in the loft)*”, explained a middle-aged Hutsul woman. The dried mushrooms were regarded as the tastiest, and in the past they were often used as a food supplement in times of food scarcity. Mushrooms were also mentioned as a food for fasting in winter before Christmas by both Hutsuls and Romanians in Ukraine. Different species of *Russula* were mentioned as good for immediate deep-frying in a pan, which “*tastes like meat*,” highlighted a Hutsul man born in 1965. Respondents distinguished *Russula* species by the colours of their caps – the tastiest had green caps, followed by light blue and purple, while the least tasty had reddish caps; red- and yellow-coloured caps indicated bitter and poisoned mushrooms. Soups and stews with dried mushrooms were called “*proper*” winter foods.

**Fig. 8:**
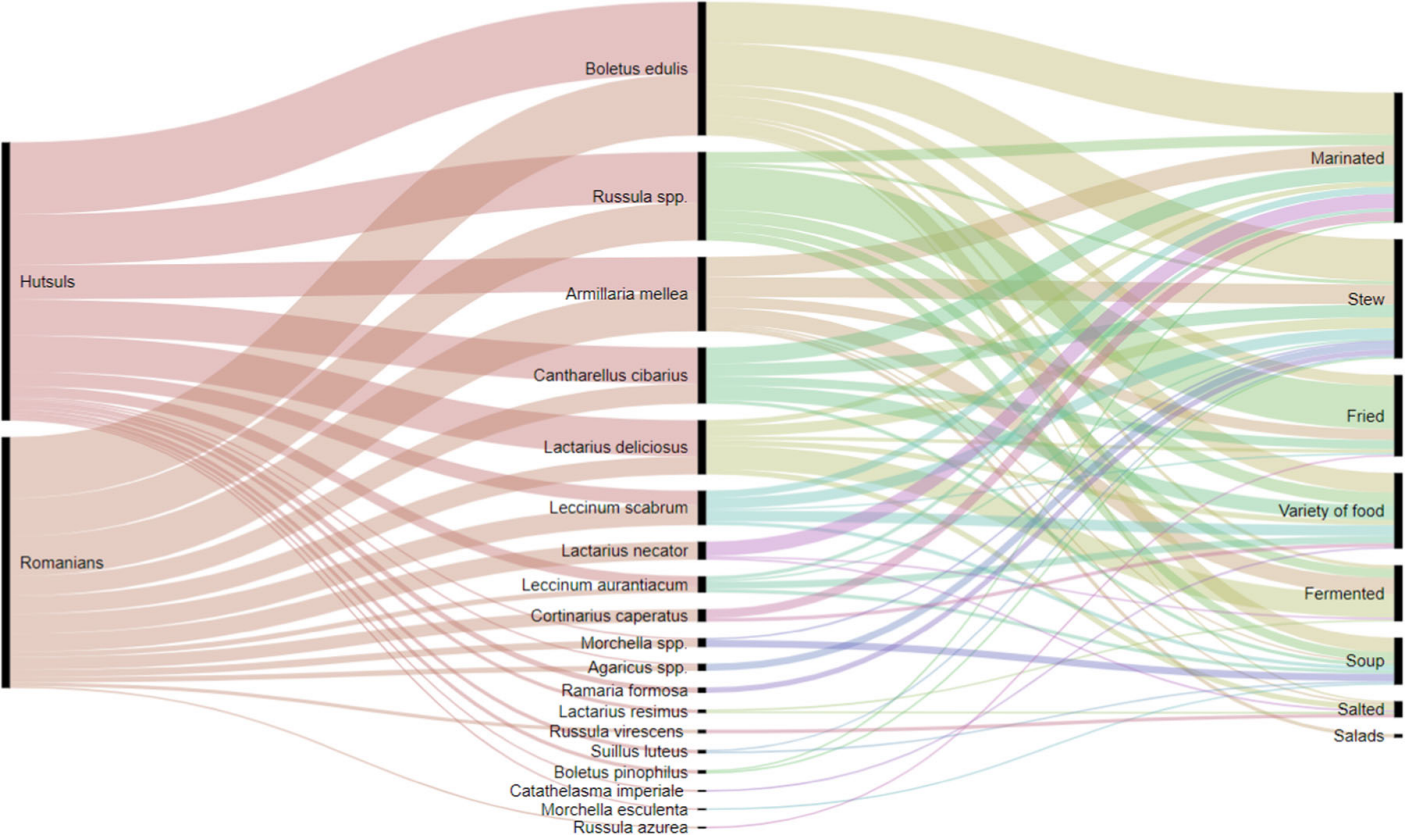
Mushroom preparations used by respondents in Ukraine

Fermented mushrooms have traditionally played important role in wintertime, although interviewees mentioned that their use is a declining practice. Pre-cooked (boiled) *Armillaria mellea* was popular among Hutsuls and Romanians in Ukraine as a seasoning for fermented cabbage. The recipe calls for a layer of boiled *Armillaria mellea* and a layer of cabbage to be fermented and kept for the whole winter. The lacto-fermentation of *Lactarius deliciosus* was mentioned by 33% of Hutsuls and 67% of Romanians in Ukraine. Lacto-fermented *Boletus* and *Russula* spp. were also mentioned once and twice, respectively (see Fig. 8). Additional interviews in Kryvorivnya village, which is the “*capital*” of Hutsul ethnos, revealed the same uses of mushrooms.

### Wild Mushrooms as Food Among Hutsuls and Romanians Living in Romanian Bukovina

Almost all the interviewees in Romania reported using mushrooms for culinary purposes. We recorded the use of nine mushroom taxa among Hutsuls, of which *Boletus edulis* and *Cantharellus cibarius* were mentioned by a great majority, while *Russula* spp. and *Armillaria mellea* were reported to a lesser extent. These mushrooms were also mentioned by Romanians who named seven taxa (plus one unidentified folk taxon). Most of the people in both communities could not clearly state the specific use of mushroom recipes as they are often just stir-fried in a pan with onions (or garlic) and accompanied by sour cream, polenta, puree or a combination of these. A 45-years-old Hutsul female reported “*I dry boletus, which is the best way. When you later boil it, it smells so good. What a taste, the forest boletus!*” In addition, *Armillaria mellea* was used for the preparation of “zacusca”, a vegetable or mushroom spread common in Romania, as well as eaten in mushroom salads and with fermented cabbage (Fig. 8).

Among Romanians, many interviewees reported the use of freezers for preserving various mushrooms, and the drying of *Boletus edulis*, *Armillaria mellea* and *Lactarius deliciosus*. The same mushrooms were also prepared as “*la murat*”, a lacto-fermented dish generally made with vegetables, especially cucumbers and cabbage. Two interviewees mentioned preparing different kinds of mushrooms as meat by breading and deep-frying them (*panè*).

Generally, “*Hutsuls are more expert on mushrooms than us*,” reported a Romanian interviewee. While the number of taxa mentioned in the two communities cannot confirm this claim, we can observe a different attitude between the two groups towards mushrooms and more generally NWFPs.

### Cross-border comparison

**Fig. 9:**
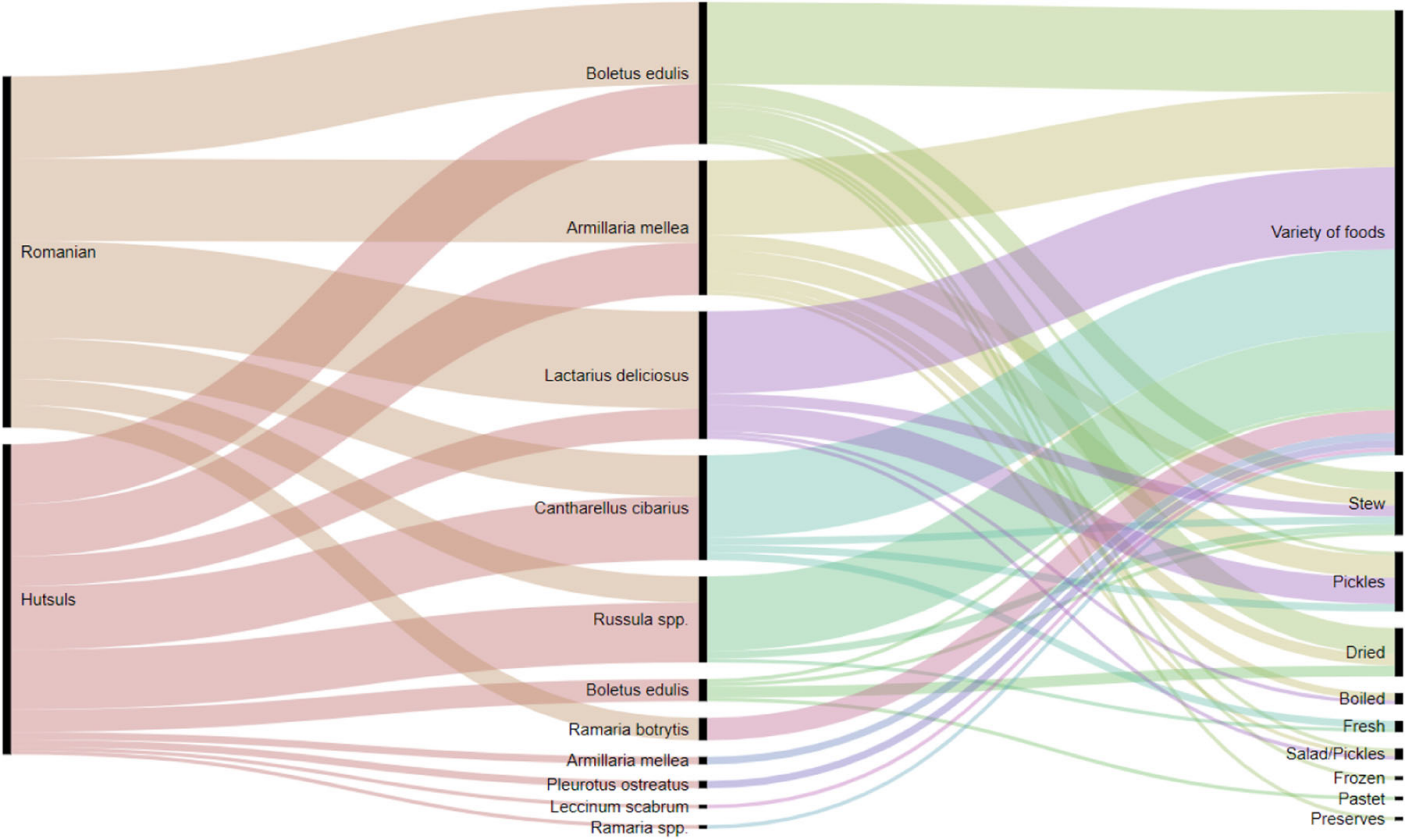
Mushroom preparations used by respondents in Romania (quite often respondents pointed out that they cook wild mushrooms as “*everyone does*” or “*regular recipe, it is obvious*”, which we recorded as “variety of foods”)

Our data (Fig. 8) shows that people in Ukraine reported more mushroom species and consistently associated these with different preparations at somewhat similar frequencies. While people in Romania (Fig. 9) reported fewer species and associated most of these with a single but mixed preparation category (i.e. “variety of foods”).

In Ukraine, due to a variety of factors, more mushroom taxa were used with greater variation, which offers a wider array of food options; however, some uses were reported by only a few interviewees. So, the TEK associated with such species could lead to the loss of that knowledge.

In Romania, people reported fewer mushroom taxa, but more consistently they referred the use of mushrooms for a “variety of foods”, which might be an indication that that knowledge is still present in the communities but used less intensely. The knowledge connected to the “variety of foods” category is also at risk of being lost if people do not transmit their knowledge. In the framework of sustainable forest management and TEK preservation, the Ukrainian cases need to be maintained, as more species and more uses are still represented here.

Quantification of the role that wild mushrooms play, calculated through the CSI, reveals that among Romanian and Ukrainian Hutsuls, as well as among Romanians living in Ukraine, *Boletus edulis* predominated as the most culturally salient taxa. However, for Romanians in Romania *Lactarius deliciosus* was the most culturally important, followed by *Boletus edulis* and *Armillaria mellea*, which have the same index value (Table 5).

**Table 5:** Cultural Significance Index (CSI) of mushrooms collected in the study areas

Taxa	Ukraine		Romania	
	Romanians	Hutsuls	Hutsuls	Romanians
*Agaricus* spp.	18	3	0	0
*Armillaria mellea*	2860	1980	1540	2200
*Boletus edulis*	5775	6510	3240	2200
*Boletus pinophilus*	0	4	0	0
*Cantharellus cibarius*	720	1620	675	396
*Catathelasma imperiale*	0	2.5	0	0
*Cortinarius caperatus*	210	0	0	0
*Lactarius deliciosus*	1200	1170	66	2328.75
*Leccinum aurantiacum*	90	270	0	0
*Leccinum scabrum*	600	420	4.5	0
*Morchella* spp.	0	0	4.5	0
*Ramaria botrytis*	0	0	6	30
*Ramaria formosa*	0	24	0	0
*Russula azurea*	10	0	0	0
*Russula* spp.	1680	3960	1120	252
*Russula virescens*	10	0	0	0
*Suillus luteus*	0	12	0	0

While *Russula* spp. was important for Hutsuls and Romanians in Ukraine and Hutsuls in Romania, *Cantharellus cibarius* was appreciated by both communities of Hutsuls and Romanians in Ukraine, but not by Romanians in Romania, which might be explained by low availability and the “hard to find” factor. Therefore, *Leccinum* spp. was not appreciated by either Hutsuls or Romanians in Romania. At the same time, *Ramaria botrytis* was popular only in Romania.

### The role of Wild Mushrooms in Local Economies

#### Wild mushrooms play a paramount role among Hutsuls living in Ukraine

**Fig. 10:**
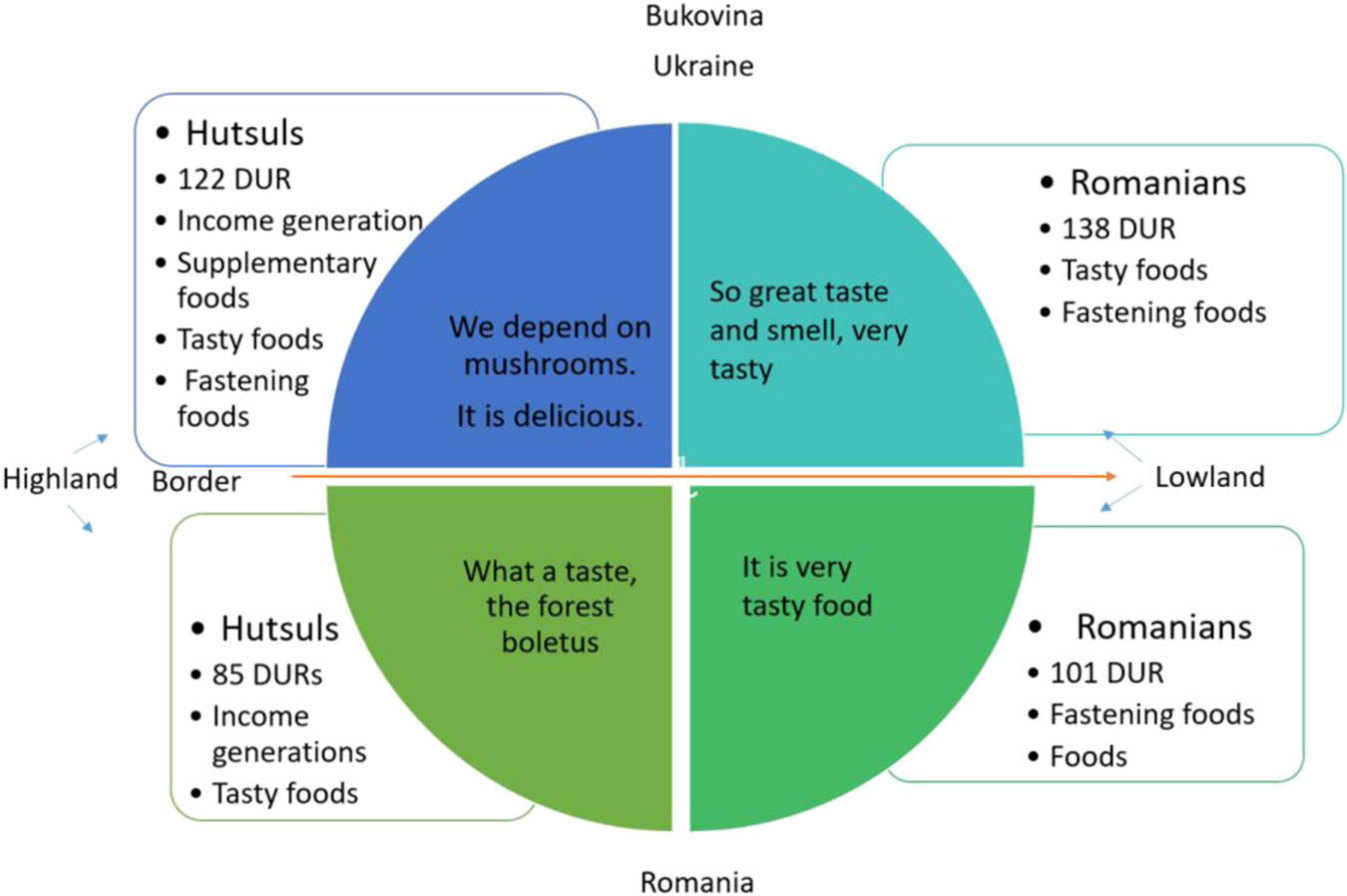
Categories of mushroom use by the four groups in Bukovina

In Ukraine, Hutsul interviewees explained that wild mushrooms are not only free foods, but also a source of income for them during times of unemployment and socio-economic crisis (Fig. 10).

These cash saving and income generation functions were seen as the most important (for some interviewees, the only source of cash income was the selling of mushrooms). “*The mushrooms and a cow are the source of living for us*,” explained a 26-year-old Hutsul man. The availability of wild mushrooms in forests makes them easy to collect in great quantities. Locals can bring the harvested mushrooms to nearly every village and sell them to collectors, usually an organization or a person that buys mushrooms from locals and then re-sells them. The most expensive mushrooms are *Cantharellus*, which can fetch a price of 150 UAH (5 EUR) per kg. The price depends on that year’s yield (the greater the yield the lower the price) as well as the quality of the mushrooms (lower quality, lower price). The buyers of *Boletus edulis* have 3 categories of prices: 1) the smallest ones with no worms, 2) bigger ones with no worms and 3) big caps possibly with worms. The prices for 2018 were 30 UAH (1 EUR) per kg for the 1^st^ category and about 15 UAH (0.5 EUR) per kg for the 3^rd^ category. All other mushroom species sold for 10 UAH per 1 kg (0.33 EUR). But, for example, in 2019 there was a very low mushroom yield, and so the price for 1 kg of *Boletus edulis* reached 120-150 UAH (4-5 EUR) per 1 kg.

During the interviews, three Ukrainian Hutsuls offered to sell us mushrooms, including a young woman who promoted her mushrooms by proclaiming “these are good mushrooms, with no worms, they are small and beautiful”. Another two interviewees showed us the *Boletu*s and *Cantharellus* mushrooms that they were going to sell to companies.

In the region, there is a factory in Putyla called “Phitofabrika” that buys wild products (such as berries, mushrooms and medical herbs) from locals and then produces different kinds of jams from them, as well as dried *Boletus* and *Cantharellus*, marinated *Boletus* and a tincture with *Cantharellus*. This is an example of the processing of wild products on the ground, providing both employment to locals and additional income for collected forest goods. However, the largest quantity of raw mushrooms was sold to re-sellers that freeze and transport them to other regions of Ukraine and abroad.

Hutsul interviewees were worried that their customary right to collect mushrooms will be suppressed. “*They (the State) created a national park, but who is it good for? Or maybe they will not allow us to collect mushrooms and berries. They will take away our last option to earn*,” stated a retired Hutsul man. Also, “*will they (the State) forbid us to collect mushrooms…*,” questioned a young Hutsul man, if the governance of wild products changes. As for Romanians in Ukrainian Bukovina, none of the interviewees feared the prohibition of mushroom picking, as they see it as a customary right and an obvious individual right to collect these forest goods.

#### Wild mushrooms play a complementary role among Hutsuls living in Romania

ROMSILVA, the National Forest Authority of Romania, reported that Suceava County is first in terms of quantity of sold mushrooms. Particularly, the most valued by the market is *Boletus edulis*, followed by *Cantharellus cibarius* and *Armillaria mellea*. Other mushrooms do not appear to hold commercial value. While both Romanian and Hutsul interviewees mentioned the possibility of selling mushrooms, we found only three Hutsul individuals that actually buy and sell them. These traders also buy several wild products from locals living in very remote areas and sell them in a town around 2.5 h away by car. Generally, the local collectors call them the night before to inform them that they have some products to be picked up. The traders drive to the collectors’ homes, weight their goods and pay them according to the daily rate, and at the end of the day deliver them to some factories in Câmpulung Moldovenesc. This town is considered the capital of this kind of trade and there are many collecting points. In summer 2019, we visited an organic certified factory of NWFPs whose owner showed us their refrigerators and driers as well as the preparation chains for international distribution (especially to Germany and Italy). Indeed, while a kilogram of *Boletus edulis* is often bought for less than 2 EUR from the person who harvested them, it can easily fetch 25-30 EUR in other European markets.

During one interview we witnessed the sale of *Cantharellus* to a reseller. In addition to money, the interviewee received food products, as they lived in a very remote settlement without a food shop. In this case, the reseller of mushrooms plays a socio-economic role by helping locals to meet their everyday needs. Hutsul interviewees reported that, in addition to local traders, during the peak season foreign enterprises come with refrigerated trucks to the upper valley and buy mushrooms or have specialized people pick them in the local forest. However, many local inhabitants considered this source of income as supplementary, which can be useful, but not their primary income. Among Romanians, this income was even less important, as mushrooms were somehow less identitarian, as their collection often requires a trip to the mountains (approximately 30 min by car) and many people reported not having the time. *“I have no time; in the last years I have not been because the children are no longer at home*,” claimed a middle-aged Romanian informant.

Finally, the different attitude of Romanians towards mushroom picking and consumption may be due to the different ecological environment (lower altitude and lower proportion of forests), as a male interviewee explained: “*There are really few mushrooms; here they do not produce a lot. There, in La Palma [in the mountains where Hutsuls live], there are people who go to the mountain and come down with strawberries, blueberries, mushrooms, every kind of wild product*.”

### Ethnomycological Knowledge is Transmitted from Generation to Generation

In all four groups in Bukovina, older generations in the family were named as a source of knowledge regarding wild mushrooms. A Hutsul man (born in 1958) explained that he learned from his parents: “*We survived because of mushrooms; we collected them when I was a kid*.” Many respondents named grandparents as teachers of how to identify edible mushrooms and their habitats. A Romanian man in Ukraine pointed out: “*I learnt from my grandma, we used to go to the forest together*.” Likewise, a Hutsul couple in Northern Bukovina explained that both of them learnt from their mothers and grandmothers. Only one Romanian man in Ukraine explained that he learned from his job, because he worked in the mountain as a forest ranger.

The general trend in all four groups was to learn from family members how to distinguish mushrooms. The dominant form of knowledge transmission was from older women, as grandmothers and mothers were often mentioned as a source of mycological knowledge. This may be explained by the fact that men were mostly away from home earning money (e.g., working abroad or at different jobs), while women took care of the house, cattle, gardens and children.

Interviewees claimed that the massive collection of the mushrooms to sell for profit was unsustainable. For example, the technique of pulling out *Boletus* mushrooms does not damage the mycorrhiza, but for commercial purposes locals do not have time for this technique and thus they simply cut the mushrooms with a knife and the rest of the mushroom bodies are left to rot, which does damage the mycorrhiza. Locals also complained that there is overharvesting of mushrooms due to their high economic value and they feared that it will negatively affect future yields; as a retired Hutsul man explained: “*They collect and sell everything and cut mushrooms instead of pulling them, which is bad and will damage future yields*”.

All of our interviewees (apart from two people) had knowledge regarding how to identify the correct mushrooms species to collect and how to prepare them. This practical knowledge also included diverse types of preparation methods and winter preserves. Hutsuls have many beliefs connected to nature and the natural environment, including mushrooms. For example, there is a strong belief that when there is a big mushroom yield a catastrophe will follow (such as war, revolution or an extremely cold winter), as a Hutsul woman (born in 1960) explained. In all four groups, there was also the belief that boiling will remove the poison from mushrooms.

**Fig. 11:**
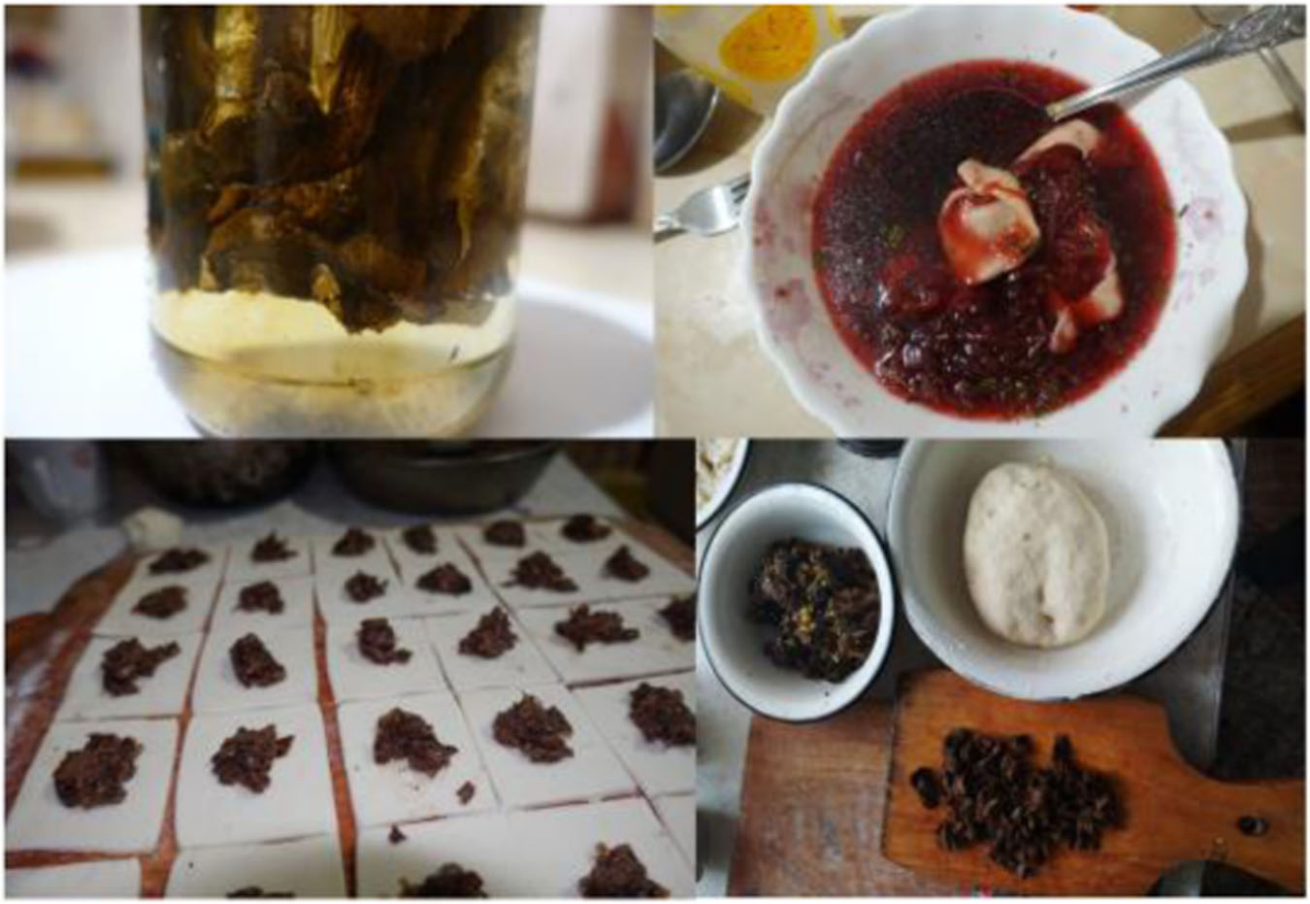
Local foods made with dried wild mushrooms: (upper pictures) dried mushrooms left in water overnight before cooking in stew with sour cream, garlic, onion and oil; (lower pictures) preparation of “vushka” for Christmas Eve. Photos by N. Stryamets, 2018-2019

Our findings reveal that wild mushrooms were used by all four groups living at the Romanian-Ukrainian border, although they play different roles in their livelihoods. In both countries, wild mushrooms play a part in direct household consumption, and therefore as a cultural attribute, e.g. food for Christmas, and fasting food (the fasting period is defined as the time of the year when Orthodox believers are not allowed to consume meat and dairy products; mushrooms were named as obligatory for those periods, “*it is good food for post [fasting time]*”, as they are a good substitute for meat, “*those Russula fried with eggs taste like meat*”) (Fig. 11).

## Discussion

Our results demonstrate that the appurtenances of an ethnic group could affect the use of wild mushrooms. The socio-economic situation in a same historical region, but divided by border, have resulted in a different extent to which people living in Ukraine and Romania depend on wild mushrooms.

### Mushrooms Play An Important Economic Role Among Hutsuls Living Across the Borders

Cash generation and direct supplemental income were highlighted among Hutsuls, but not among Romanians in both countries. For Hutsuls in Ukraine, wild mushrooms play the role of a safety net as an additional food resource in case of food scarcity. Mushrooms also provide secondary, or even in some cases primary, employment for Hutsuls in Ukraine, in contrast to only seasonal supplementary cash income for Hutsuls in Romania. The dependence on wild mushrooms as source of additional income are also demonstrated by other studies (Bonet et al. 2014, Peintner et al. 2013, Stryamets et al. 2015). Our data shows that both ethnos are mycophilic. However, traditionally Hutsuls have been more connected to the collection of mushrooms.

Hutsuls, often defined as “*forest people*”, have used forest products for centuries; and historical data shows that mushrooming was the responsibility of women and children (Vagilevich 1855). Having limited arable land resources and a harsh and cold climate, Hutsuls were traditionally gatherers of forest products, with a large quantity of mushrooms collected. Mushrooms not only play the role of a dietary supplement but they were also used in ritual foods (Kozholyanko 2015). Hutsuls themselves said, “Пішов в гриби” – “*I went to the mushrooms*”. Not to collect mushrooms, but to be with the mushrooms, which can perhaps be explained as there is such an abundance of mushrooms that one feels among them. Romanians living in the lowlands of the Carpathian Mountains have enough arable lands, and mostly engaged in small-scale agriculture, while forest products were used only occasionally. Interviewees living at lower altitudes, in pre-Carpathian lowlands stated “*Oh, mushrooms, you need to go to the Carpathian Mountains for mushrooms, and ask Hutsuls*”, and also “*You need to get up early to reach the forest or use a car to get to the forest to collect mushrooms*”.

According to Ward et al. (2018), the factors that affect the use of wild mushrooms include formal and informal institutional laws, physical assets, social relationships, identity, and knowledge. In our case studies, both Ukraine and Romania provide free access to wild mushroom collection in state-owned forests (formal institutional laws). This also applies to the past, before 1940, when the same formal institutional laws applied to all four groups. Customary rights for the collection of mushrooms are strongly established in both parts of Bukovina (informal institutional laws). The cross-border analysis shows that access to local resources is the same, but economic conditions are different due to different political situations. The physical assets are also the same. Likewise, Ukrainian and Romanian Hutsuls, as one ethnic group, have the same identity. However, after the division of Bukovina between Ukraine and Romania, the socio-economic situations of the two communities changed, and this has influenced the use of wild mushrooms. The economic crises of the 1990s in Ukraine created the dependence of locals in rural areas on wild mushrooms. This occurred as a consequence of the drop in GDP caused by collapse of the Soviet Union. Indeed, in this time expenses for food accounted for a large proportion of the average household expenditure (Nello 1992). However, most of the people survived thanks to both wild resources and small cultivated patches (dachas) (Sõukand et al. 2020). Indeed, several scholars agree that studies NWFPs play a role of safety net both as food and as a possibility of gaining income in times of crisis (Janse and Ottitsch 2005, Shackleton and Pandey 2014, Steele et al. 2015).

Currently, the enhancement of the economic context resulted in a flourishing development of the market of wild mushrooms both nationally and internationally (de Frutos 2020). Wild-collected mushrooms are more highly appreciated and valued than cultivated ones (Voces et al. 2012). National demand is the result of the popular use of wild mushrooms during the winter holidays (e.g. Christmas, fasting period) (Kozholyanko 2015). Mushrooms are easy to store after drying and the value of dried mushrooms is highly appreciated, especially in towns and as an export abroad, as in Europe there is growing demand for wild mushrooms which are collected in wild ecosystems (Voces et al. 2012). In remote mountain areas of Ukraine, which are still struggling to fight unemployment and poverty such as Putyla (the poorest district of the Chernivci region (Anon 2018), people make profit of all available means and especially on NWFP including wild mushrooms as they are free and easy to obtain resource. Indeed, in Northern Bukovina locals have free access to the market and there are no restrictions, and even though a permit for the commercial use of mushrooms needs to be obtained, only companies that export mushrooms to European countries obtain such a permit (Stryamets et al. 2019). Locals also sell marinated and dried mushrooms at babushkas markets (cf. Sõukand et al. 2020), which is a way to preserve those practices, while in Romanian Bukovina at the markets were not observed such practices. This is probably because Southern Bukovina, as a part of the European Union, enjoys a better socio-economic situation in terms of social benefits, employment and subsidies for local farmers, which renders mushrooming a recreational, not a subsistence, activity. Therefore, during fieldwork in both areas of Bukovina, the selling of wild mushrooms to resellers was observed, but more in the Ukrainian part. At the same time, Hutsuls living in Ukraine expressed their fear that overharvesting and not “proper harvesting techniques” would result in the future reduction of mushroom harvests.

### Hutsuls and Romanians Show A Different Attitude Towards Collecting Mushrooms

Cross-cultural comparison revealed (Pacheco-Cobos et al. 2015), that Hutsuls are people of the forest, live closer to the forest in remote areas and rely more on local resources for both their nutritional, cultural and spiritual needs. This suggests that the use of wild mushrooms as part of NWFPs, is an expression of the local cultural landscape, which is in line with that suggested by Plieninger et al. (2013). The cultural importance of collecting NWFPs was more important than their actual nutritional value for many of our interviewees.

Cultural significance index analysis shows that there is a cross-border difference. *Boletus edulis* and *Boletus* spp. are important for Hutsuls in both areas and Romanians in Ukraine, which is one of the most popular mushroom taxa in the world (Boa 2004). The popularity of *Lactarius* in Romanian Romanians is hard to trace as it was reported as popular by both Ukrainians living in Maramures and other Romanians (Łuczaj et al. 2015, Tanase and Grudnicki 2003). *Leccinum* spp. popularity in Ukrainian Bukovina (compare to almost no uses in Romanian Bukovina) could be explained by the popularization of mushrooming in schools and during Soviet times.

Hutsuls have more knowledge on species and more diverse uses. But for Romanians, living far from forests, use of wild mushroom have less diversified, but still active. For both Hutsul communities, mushroom collection plays a role in income generation and functions as a safety net, while among Romanians it is more a leisure time activity.

Among both Romanians and Hutsuls, wild mushroom collecting is a cultural and family activity, as a Hutsul men from Ukrainian Carpathians highlighted *“We go together with my son to collect mushrooms, we spend half of a day, taking sandwiches and having nice time”*. Traditional foods were mentioned by all four groups as important for certain periods of the year and holidays. The traditional ways preserving mushrooms for winter by drying or fermenting them are been influenced by the new practice of freezing, which was also observed for example in Roztochya, Ukraine (Stryamets et al. 2012) and Poland (Łuczaj and Nieroda 2011). Diversification of the local diet and “*free*” foods were named by all four groups as well.

Towards the knowledge transmission pattern, our results show more static from grandparents and parents. In the same region, the same ethnic groups towards the use of medical plants show the divergent and influenced by books knowledge in Ukrainian part (Mattalia et al. 2020, 2021), or food uses of wild plants influenced by intermarriage and social interactions (Stryamets et al. 2021). This might be explained that the use of mushrooms has more traditional uses as they could be poisonous.

The mushroom picker profile in all four groups was similar, involving elderly people and children, and slightly more men, which was also the case in the mountain region of Poland (Łuczaj and Nieroda 2011). Knowledge of mushrooms is the domain of both sexes same as for example in Mexico (Ruan-Soto 2018)., but the activities of cooking and preserving mushrooms are dominated by women like in Poland for example (Łuczaj and Nieroda 2011), while in Sweden is cooked by both sexes (Stryamets et al. 2012).

In Northern Bukovina, locals have free access to the market and there are no restrictions, and even though a permit for the commercial use of mushrooms needs to be obtained, only companies that export mushrooms to European countries obtain such a permit (Stryamets et al. 2019). Locals also sell marinated and dried mushrooms at babushkas markets (cf. Sõukand et al. 2020), which is a way to preserve those practices, while at the markets in Romanian Bukovina such practices were not observed.

### What can we learn from this study? - Wild mushrooms can contribute to a sustainable rural development in the Carpathians

Nerfa et al. (2020) proposed to distinguish between absolute and relative forest income: the former refers to the sum total of the monetary value of products harvested from the forest, while the latter refers to the proportion of household income comprised by forest income. In our Ukrainian sample, many Hutsul interviewees viewed forest and forest products as absolute forest income. However, Romanian interviewees highlighted that NWFPs represented a supplementary income, and so relative forest income. On the basis of this finding, decisions regarding absolute forest income should take into consideration the needs of local rural residents in marginal mountain areas.

Sustainable forest management underlines the need for the optimization of benefits, including cultural benefits, for local rural livelihoods generated from forests. The New York Declaration on Forests (UN Climate Summit 2014) stressed that forests can contribute to economic growth, poverty alleviation, food security and many other issues. Forest-dependent indigenous and local groups have the right to participate in the decision-making process regarding forest management (Garibay-Orijel et al. 2009). Forest governance should be strengthened and transparent, and indigenous and local people given the right to participate in the forest management decision-making process (Stryamets et al. 2019). For example, the creation of new protected areas in Ukraine causes people to fear the restriction of access to wild mushrooms, as according to legislation in strict protected reserves, as well as in core areas of national parks, access to the collection of wild mushrooms is forbidden (Anon 1992).

Wild mushrooms currently play an important role on both sides of the border; however, the level and manner of dependence on mushrooms are different. Our findings demonstrate that for mountain communities in Bukovina the use of forests products is crucial in terms of income generation, food supply and cultural importance, and therefore decision makers should consider the needs of locals. The decision-making process should take into account the high value that forest products have for locals, especially when establishing new protected areas, which might limit access to resources. Although the food use of mushrooms was highest among Hutsuls in Ukraine, we found that the use of wild mushrooms depends more on the socio-political and economic situation in a country, and less on belonging to a specific ethnic group. The cross-cultural analysis emphasises the different CSI in fungal diversity and importance. The cross-border analysis also proves that governance and the economic situation in the country have direct impact on the use of wild fungi.

Our findings suggest that mushrooms use should be included in multiple-use forest management planning, which has to ensure that timber and other forest products, like mushrooms, are managed in a complementary manner.

## Conclusions

Our results demonstrate that the appurtenances of an ethnic group could affect the use of wild mushrooms. Romanians living in lowlands far from the forest and with limited forest resources used the same species of mushrooms and with the same preparations, but they did not sell them. Hutsuls on both sides of the Ukrainian-Romanian border used mushrooms to generate income, but Hutsuls in Southern Bukovina mentioned them as a “life-saving resource in the past”, while in Northern Bukovina they remain a “precious resource” today. We can conclude that the economic situation in the two countries plays a crucial role in the use of wild mushrooms. The reasons for collecting mushrooms differ among our interviewees: recreational, cultural and diversification of the diet among Hutsuls in Southern Bukovina, and cultural and economic (cash generation) among Hutsuls especially those living in Northern Bukovina. In our Ukrainian sample, many Hutsul interviewees viewed forest and forest products as absolute forest income, while Hutsuls in our Romanian sample highlighted that they were a supplementary income. On the other hand, for Romanians in both areas wild mushrooms represented a source of diversification of the local diet.

The use of wild mushrooms should be integrated into sustainable forest management models for indigenous and local communities, including multiple-use forest management planning, which must ensure that timber and other forest products, such as mushrooms, are managed in a complementary manner. The local processing of forest products provides both job opportunities and value-added products for regional and local markets. Future research should focus on the cultural and especially ritual use of wild mushrooms, which was highlighted by interviewees in all four groups.

## Data Availability

All data are available in this publication.
